# Rehabilitation for People Living with Dementia: A Scoping Review of Processes and Outcomes

**DOI:** 10.1155/2019/4141050

**Published:** 2019-06-02

**Authors:** Maiken B. Ravn, Kirsten S. Petersen, Jette Thuesen

**Affiliations:** ^1^Department of Health, Science, and Technology, Aalborg University, Aalborg, Denmark; ^2^REHPA, Danish Knowledge Centre for Rehabilitation and Palliative Care, Nyborg, Denmark

## Abstract

**Objectives:**

The aim of this scoping review was to map intervention studies of rehabilitation for people living with dementia regarding processes and outcomes, with a particular focus on whether the intervention is person-centred, home-based, or organised adopting a multidisciplinary approach and measures outcomes relating to everyday functioning and well-being.

**Methods:**

A systematic search of electronic databases was conducted in PubMed, CINAHL, PsycINFO, Embase, and Cochrane. Studies from 2005 to November 2018 were collected and screened for relevance and quality. Randomised control trials and prospective cohort trials reporting a statistically significant effect on one or more outcome measures were included. Included studies were mapped according to selected processes and outcome measures.

**Results:**

Twenty-six intervention studies were included and mapped. Nineteen of the interventions were person-centred, nine were home-based, and 14 reported a multidisciplinary approach. Twelve of the interventions had activities of daily living as an outcome measure, and 14 had quality of life as an outcome measure.

**Conclusion:**

Person-centredness appears in most rehabilitation interventions for people living with dementia. Other processes and outcomes are heterogeneously described in the research literature. Rehabilitation programmes can be home-based or take place at a centre. Although not exclusive, the organisation of rehabilitation can be multidisciplinary. Fewer than half of the intervention studies measure the impact on activities of daily living and quality of life. Future guidelines must take into account the weak evidence regarding these aspects.

## 1. Introduction

Dementia is one of the main causes of disability and dependency among elderly people worldwide [1–3]. The World Alzheimer Report from 2015 estimates that 131.5 million people worldwide will be living with dementia in 2050 [4]. The understanding of dementia has changed over time, and more recently, a biopsychosocial approach has been applied [5, 6] in which it is discussed whether dementia should be considered a disability [7]. Consequently, there is an increasing focus among clinicians, politicians, and people living with dementia (PLWD) and their families on how to improve and maintain daily functions and slow a person's decline into dementia [2, 8].

Like other European countries, Denmark has designed a national action plan for dementia which involves rehabilitation [9, 10]. Also, rehabilitation appears as a core recommendation in the recent World Health Organisation (WHO) global action plan on the public health response to dementia [11]. Rehabilitation is increasingly recognised as contributing to dementia care, both as a practical framework and as a guiding philosophy [10, 12–16]. This emphasises the need for comprehensive programmes regarding rehabilitation for PLWD [16–18]. This review is part of a more comprehensive study developing comprehensive rehabilitation interventions for PLWD in Denmark, conducted by the Danish Knowledge Centre for Rehabilitation and Palliative Care, REHPA.

According to the Medical Research Council (MRC), developing complex interventions such as comprehensive rehabilitation requires the identification of evidence, identifying or developing theory, and modelling processes and outcome [19]. Providing evidence in rehabilitation for PLWD is challenged by a lack of conceptual consensus [16–18] and by heterogeneity regarding samples, rehabilitative techniques, and the processes and outcome measures used in intervention studies [18, 20]. Several studies indicate that multimodal nonpharmacological interventions are promising [18, 20–23]. However, evidence concerning which modalities should be integrated into a comprehensive rehabilitation intervention in dementia is poor [16, 18, 20]. Evidence for specific modalities has been identified: physical training [24, 25], memory training [26, 27], occupational therapy [28], dyadic interventions [29], and cognitive stimulation therapy [30]. Moreover, there is a small but growing evidence base regarding the effectiveness of cognitive rehabilitation (CR) [12, 31–33]. CR includes a variety of psychosocial interventions aiming to support functioning, participation, and family carers [12, 31–33].

In accordance with the MRC guidance, this review will focus on selected aspects regarding modelling processes and outcomes in dementia rehabilitation for PLWD. The research literature concerning processes and outcomes seems to be heterogeneous. Professor Linda Clare and colleagues emphasise the significance of a person-centred approach tailored in accordance with individually meaningful goals and further emphasise the significance of the setting in rehabilitation. According to Clare, CR aims to support aspects of everyday functioning and well-being [31]. Clare does not clearly address whether CR should be organised by a multidisciplinary approach. This is stressed by other researchers in rehabilitation for PLWD [17] and further supported in generic rehabilitation literature [34, 35].

The aim of this scoping review is to map intervention studies of rehabilitation for PLWD regarding processes and outcomes with a particular focus on whether the intervention is person-centred, home-based, adopts a multidisciplinary approach, and measures the outcome on everyday functioning and well-being.

## 2. Materials and Methods

The scoping review was undertaken to identify the nature and extent of rehabilitation interventions targeting PLWD and further analyse processes and outcomes [36]. The screening process followed the PRISMA Statement and Guidelines [37, 38].

### 2.1. Search Strategy

Relevant articles were retrieved from PubMed, CINAHL, PsycINFO, Embase, and Cochrane electronic databases from 2005 until the 11th of November 2018. The year 2005 was chosen because a Cochrane review found no studies regarding cognitive rehabilitation in 2003 [33], and two books indicated an emerging focus on rehabilitation in dementia in 2005 [15] and 2007 [31]. Search terms used included (Alzheimer's disease [Title/Abstract] OR “Alzheimer Disease” [Mesh] OR “Dementia” [Mesh:NoExp] OR dementia [Title/Abstract] OR senile) AND (Rehabilitation [Mesh:NoExp] OR rehabilitation [Title/Abstract] OR reable^*∗*^) NOT (brain injury OR stroke [MESH Major Topic] OR surgery NOT down syndrome OR postoperative OR “Postoperative Period”[Mesh:NoExp]). The search was adopted to the individual databases. The search terms “Dementia” and “Alzheimer Disease” are broad MESH terms including “Lewy Body Disease,” “Vascular Dementia,” and “Frontotemporal Dementia.” The search strategy was developed in cooperation with an experienced librarian and through inspiration from previous systematic reviews [24, 39, 40].

Mendeley Desktop Version 1.17.8 was used to manage the retrieved articles and remove duplicates. Furthermore, experts within the field of dementia and rehabilitation research were consulted regarding the research strategy and the preliminary findings.

### 2.2. Inclusion and Exclusion Criteria

The eligible studies were screened based on the type of article, population, intervention, outcome, and study design. Studies were excluded if not all eight criteria were applied: (1) peer-reviewed articles in English, Danish, Swedish, or Norwegian, (2) the term “rehabilitation” used in title/abstract/keywords, (3) dementia (Alzheimer's disease or not specified) being the primary diagnosis, (4) mild to moderate dementia, (5) participants were home-dwelling, inpatients, or intermediate care patients, (6) the intervention addressed more than one International Classification of Functioning, Disability and Health (ICF) component: body system and function, activities and participation, and environmental factors [41], (7) intervention studies with a statistically significant effect on one or more outcome measures, and (8) high level of evidence intervention studies (prospective cohort and randomised controlled trials) [42].

Rehabilitation is, in this review, defined according to the biopsychosocial model [5, 6]. Hence, interventions targeting only one ICF component was not considered as rehabilitation and thereby excluded. By including only intervention studies with a high level of evidence and a statistically significant effect on one or more outcome measures, this review aimed to map the most promising intervention studies in rehabilitation for PLWD.

### 2.3. Study Selection

**Figure 1:**
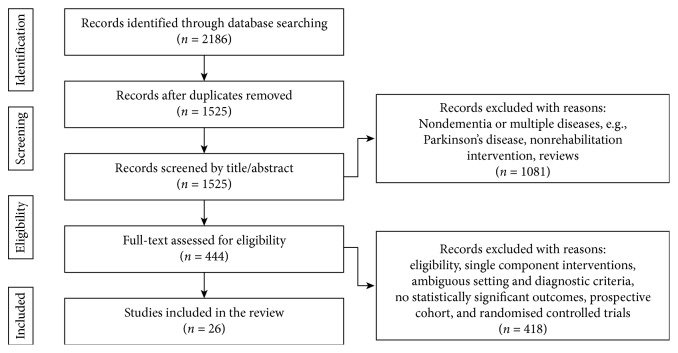
PRISMA flow diagram, identification including inclusion and exclusion of studies.

The first screening of title and abstract was undertaken by the first author and, if in doubt, articles were included and discussed with the other reviewers. The main reason for exclusion was dementia (Alzheimer's disease or not specified) not being the primary diagnosis. Included studies were screened for eligibility; the full-text articles were read and analysed by all authors. When in doubt, a consensus was reached by discussion. The main reasons for exclusion were studies in which interventions addressed only one ICF component and/or the setting and diagnostic criteria were ambiguous. The flowchart of the search and screening process is presented in Figure 1.

### 2.4. Quality Assessment

A quality assessment of the included studies was performed using CONSORT for randomised control trials and CASP for prospective cohort studies. The assessment of included studies did not result in further exclusion.

### 2.5. Analytic Framework for Mapping

The interventions in the included studies were mapped by all authors according to the study's aim. The following aspects of the included studies were predefined: *person-centred* is defined as interventions that are either tailor-made, individualised, or involving the participants' own goals as person-centred rehabilitation involves both organisation and delivery [12, 34]; *home-based* interventions are interventions taking place in the homes of people living with dementia; *multidisciplinary* is considered as interventions that include two or more professions; *everyday functioning* is defined as activities of daily living (ADL) as an outcome measure, according to the Danish Dementia Research Centre's list of ADL-scales relevant in dementia research and scales explicitly termed as activities of daily living [43]; and *well-being* is defined as quality of life (QoL) as an outcome measure, according to JPND research's list of QoL-scales, and scales explicitly described as quality of life [44].

Two authors independently mapped and compared their assessment of the interventions reported in the included studies. In the event of any disagreements, a third author was consulted until consensus was reached.

#### 2.5.1. Patient and Public Involvement

No patients were involved in the review process. Peers were involved in planning the review via discussions with fellow researchers in a research network.

## 3. Results

The systematic bibliographic database search identified 2,186 potentially relevant abstracts, a total of 1,525 after merging duplicates. Following the first screening of title and abstract, 1,081 abstracts did not meet the inclusion criteria. Full-text reading was undertaken with the remaining eligible 444 studies, of which 26 studies met the inclusion criteria and were included in this review.

Nineteen of the 26 interventions were person-centred, two were not specified, and five were not considered to be person-centred. Nine of the 26 interventions were home-based, six were not specified, and 11 were not home-based. Fourteen of the 26 interventions reported a multidisciplinary approach towards rehabilitation, and 12 did not. Twelve studies had ADL as an outcome measure. Fourteen studies had QoL as an outcome measure.

As an additional finding, we realised that the 13 studies termed as “cognitive rehabilitation” differed on several of the mapped aspects. For example, seven were multidisciplinary, six were not; four were home-based, six were not home-based, and three were not specified; 11 were person-centred, one was not person-centred, and one did not specify person-centredness. Details regarding the mapping of the 26 studies are presented in Table 1.

## 4. Discussion

The aim of this scoping review is to map intervention studies of rehabilitation for PLWD regarding processes and outcomes, with a particular focus on whether interventions are person-centred, home-based, adopt a multidisciplinary approach, and measure outcomes relating to everyday functioning and well-being. The findings provide an overview of the current evidence base in which these specific aspects are presented in statistically significant intervention studies of rehabilitation for PLWD. Nineteen of the 26 interventions were person-centred, that is, tailor-made, individualised interventions or interventions involving the participants' own goals. This aligns with generic rehabilitation literature: a person-centredgoal-orientated rehabilitation has been recommended by several well-cited researchers [71, 72]. Moreover, person-centredness is a well-known principle in dementia care [73]. However, the review identified a few effective interventions which did not address person-centredness [56, 58, 64, 67, 68]. Thus, the results of this review support person-centredness as a dominant aspect within rehabilitation in PLWD.

Regarding the setting of the interventions, in nine of the 26 included studies, the intervention was home-based. Within generic rehabilitation literature, Professor Derick T. Wade emphasises the importance of the environment without stating that rehabilitation should be restricted to one specific setting [74]. Home-based rehabilitation provides the opportunity for PLWD to engage in rehabilitation in their everyday setting where they spend most of their time [75]. It has been suggested that home-based rehabilitation interventions may reduce the demands and pressures on the participants [76]. Despite this benefit, the results of this review indicate that a home-based setting is not commonly used in rehabilitation for PLWD. According to our findings, rehabilitation of PLWD may take place either at home or at a centre.

A multidisciplinary approach was present in 14 of the 26 interventions studied. Studies from geriatric rehabilitation [77] and rehabilitation in other areas support a multidisciplinary approach [78]. This is reflected in other literature on rehabilitation in dementia [15, 17]. However, the results in the review illustrate that 12 intervention studies did not undertake a multidisciplinary approach; hence, the review does not clearly support multidisciplinarity as an organising principle in rehabilitation in dementia.

Fewer than half of the studies measured the impact on ADL, and more than half of them measured the impact on QoL. Dementia being an incurable and progressing disease affecting everyday life [79, 80], outcome measures related to ADL and QoL seem relevant in rehabilitation for PLWD. Furthermore, difficulties in performing ADL are associated with diminished QoL and poor self-efficacy [22]. Still, the impact on ADL and QoL is measured only in about half of the intervention studies. Thereby, the results of this review concur with the findings from Kroll and Naue indicating that there is a heterogeneous use of outcome measures in intervention studies within rehabilitation for PLWD [18].

Like the overall results, interventions termed as “cognitive rehabilitation” did not have consistency regarding whether those intervention studies for PLWD were person-centred, home-based, or multidisciplinary. Thus, the results of our review support and expand the critique referred to in the introduction, illustrating that studies in rehabilitation for PLWD suffer from a conceptual inconsistency [16–18] and substantial heterogeneity in terms of processes [18, 20].

The review has both methodological strengths and limitations. The strength of the findings from the review is the provision of an overview of the processes and outcomes in studies of high evidence in the rehabilitation of PLWD.

However, the study also has some limitations. Using search terms other than “rehabilitation” and “reable^*∗*^,” for example, “restorative care,” might have resulted in more relevant studies. However, the rationale behind this decision was to include studies constituted as rehabilitation. Furthermore, we do not expect that including more studies would have resulted in a clearer picture of processes and outcomes in dementia intervention studies.

Only studies with a high level of evidence and a statistically significant effect on more than one outcome measure were included in this review. In accordance with the aim, these studies were assessed to be the most reasonable. However, it can be argued that including studies with no statistically significant effects might have been just as reasonable. Also including studies with no statistically significant effect might have provided us with a more comprehensive picture of current evidence. Results from studies with no statistically significant effect are more difficult to publish, so risk of publication bias should be considered [81]. Considering the aim, we however do not expect this would have resulted in a clearer picture of processes and outcomes in dementia intervention studies. Furthermore, we made initial analysis including studies with no statistical significant effect and these studies did not differ from studies with a statistical significant effect. A total of four studies were excluded due to no statistical significant effect [82–85].

In the categorisation of ADL outcome measures in the studies, we did not include the Canadian Occupational Performance Measure or dual-task performance, both arguably linked to maintaining meaningful activities for PLWD [86, 87]. However, we do not expect this would have resulted in a clearer picture of processes and outcomes in dementia intervention studies either.

## 5. Conclusion

This review offers an overview of specific processes and outcomes present in intervention studies of rehabilitation for people with dementia. The review shows how the included intervention studies support person-centredness as a key aspect in rehabilitation for PLWD. Other processes and outcomes are heterogeneously described in the research literature. When it comes to the settings of the included studies, the review indicates that rehabilitation can be home-based or take place at a centre. Unlike recommendations from other sources, the review does not provide guidance regarding whether rehabilitation for PLWD should take a multidisciplinary approach. Regarding outcomes, the review illustrates that far from all, intervention studies measure the impact on ADL and QoL. Finally, the review shows that the heterogeneity of the processes and outcomes applies to the total group, as well as to the subgroup of CR intervention studies. Future guidelines must take into account the weak evidence regarding processes and outcomes.

## Figures and Tables

**Table 1:** Mapping of the included studies.

Intervention and reference	Processes			Outcome	
	Multidisciplinary	Home-based	Person-centred	ADL^a^	QoL^b^
Brain-activating rehabilitation [45]	Yes	No	Yes	No	No
Brain-activating rehabilitation [46]	No	No	Yes	No	Yes
Cognitive rehabilitation and cognitive-behavioural treatment [47]	No	No	Yes	No	No
Cognitive rehabilitation [48]	Yes	No	Yes	Yes	Yes^*∗*^
Cognitive rehabilitation [49]	Yes	No	Yes	Yes	Yes^*∗*^
Cognitive rehabilitation [50]	No	No	Not specified	No	No
Cognitive rehabilitation [51]	Yes	Yes	Yes	Yes	Yes
Cognitive rehabilitation [52]	Yes	Yes	Yes	No	No
Cognitive rehabilitation [53]	No	Not specified	Yes	No	No
Cognitive rehabilitation [54]	No	Yes	Yes	No	Yes
Cognitive rehabilitation [55]	No	Not specified	Yes	Yes	Yes^*∗*^
Collaborative memory intervention [56]	No	Yes	No	No	No
Computer errorless learning-based memory training program [57]	No	Not specified	Yes	Yes^*∗*^	No
Dual-task rehabilitation [58]	No	Not specified	No	No	No
Individualised cognitive rehabilitation therapy [59]	Yes	Not specified	Yes	Yes^*∗*^	Yes
Individualised face-to-face cognitive rehabilitation [60]	No	Yes	Yes	Yes	Yes
Intensive rehabilitation [61]	Yes	Not specified	Yes	Yes	No
MINDVital rehabilitation [62]	Yes	No	Yes	Yes	No
Multicomponent cognitive stimulation program [63]	No	Yes	Yes	No	No
Multidisciplinary cognitive rehabilitation [64]	Yes	No	No	No	Yes^*∗*^
Multidisciplinary rehabilitation [65]	Yes	No	Not specified	No	Yes^*∗*^
Multimodal cognitive and physical rehabilitation [66]	Yes	No	Yes	Yes	Yes
Multimodal rehabilitation [67]	No	Yes	No	Yes	No
Music rehabilitation [68]	Yes	Yes	No	No	Yes
Self-management group rehabilitation [69]	Yes	No	Yes	No	Yes
Short-term inpatient rehabilitation [70]	Yes	Yes	Yes	Yes	Yes

^a^Activities of daily living, according to the Danish Dementia Research Centre [43]. ^b^Quality of life, according to JPND research [44]. ^*∗*^Statistically significant effect (*p* ≥ 0.05).

## References

[1] Nationalt videnscenter for demens (2017). *Vaskulær Demens*.

[2] Scott K. R., Barrett A. M. (2007). Dementia syndromes: evaluation and treatment. *Expert Review of Neurotherapeutics*.

[3] World Health Organization (2017). *Dementia. 2017 12-12-2017*.

[4] Prince M. (2015). *World Alzheimer Report 2015 the Global Impact of Dementia*.

[5] Downs M., Clare L., Anderson E., Woods B., Clare L. (2008). Dementia as a biopsychosocial condition: implications for practice and research. *Handbook of the Clinical Psychology of Ageing*.

[6] Downs M., Clare L., Mackenzie J. (2006). Understandings of dementia: explanatory models of dementia and their implications for the person with dementia and therapeutic efforts. *Dementia: Mind, Meaning, and the Person*.

[7] Alzheimer Europe (2017). *2017: Dementia as a Disability? Implications for Ethics, Policy and Practice*.

[8] Perkins R. (2012). Evidence-based practice interventions for managing behavioral and psychological symptoms of dementia in nursing home residents. *Annals of Long Term Care*.

[9] Georges J. (2016). *2012: National Dementia Strategies (Diagnosis, Treatment and Research)*.

[10] Sundheds- og Ældreministeriet (2016). *Et Trygt Og Værdigt liv Med Demens*.

[11] World Health Organization (2017). *Global Action Plan on the Public Health Response to Dementia 2017–2025*.

[12] Clare L. (2017). Rehabilitation for people living with dementia: a practical framework of positive support. *PLoS Medicine*.

[13] Clare L. An enablement approach to dementia care and prevention.

[14] Cohen D., Eisdorfer C. (1986). *The Loss of Self: a Family Resource for the Care of Alzheimer’s Disease and Related Disorders*.

[15] Marshall M. (2005). *Perspectives on Rehabilitation and Dementia*.

[16] Poulos C. J., Bayer A., Beaupre L. (2017). A comprehensive approach to reablement in dementia. *Alzheimer’s & Dementia: Translational Research & Clinical Interventions*.

[17] Cations M., Laver K. E., Crotty M., Cameron I. D. (2017). Rehabilitation in dementia care. *Age and Ageing*.

[18] Kroll T., Naue U. (2011). The state and context of evidence production and knowledge translation in the rehabilitation of people with Alzheimer’s disease. *Dementia*.

[19] Craig P., Dieppe P., Macintyre S., Michie S., Nazareth I., Petticrew M. (2008). Developing and evaluating complex interventions: the new Medical Research Council guidance. *BMJ*.

[20] Bahar-Fuchs A., Clare L., Woods B. (2013). Cognitive training and cognitive rehabilitation for mild to moderate Alzheimer’s disease and vascular dementia. *Cochrane Database of Systematic Reviews*.

[21] Olazarán J., Reisberg B., Clare L. (2010). Nonpharmacological therapies in Alzheimer’s disease: a systematic review of efficacy. *Dementia and Geriatric Cognitive Disorders*.

[22] Orellano E., Colón W. L., Arbesman M. (2012). Effect of occupation- and activity-based interventions on instrumental activities of daily living performance among community-dwelling older adults: a systematic review. *American Journal of Occupational Therapy*.

[23] Zabalegui A., Hamers J. P. H., Karlsson S. (2014). Best practices interventions to improve quality of care of people with dementia living at home. *Patient Education and Counseling*.

[24] Pitkälä K., Savikko N., Poysti M., Strandberg T., Laakkonen M.-L. (2013). Efficacy of physical exercise intervention on mobility and physical functioning in older people with dementia: a systematic review. *Experimental Gerontology*.

[25] Forbes D., Forbes S. C, Blake C. M, Thiessen E. J, Forbes S (2015). Exercise programs for people with dementia. *Cochrane Database of Systematic Reviews*.

[26] De Vreese L. P., Neri M., Fioravanti M., Belloi L., Zanetti O. (2001). Memory rehabilitation in Alzheimer’s disease: a review of progress. *International Journal of Geriatric Psychiatry*.

[27] Creighton A. S., van der Ploeg E. S., O’ Connor D. W. (2013). A literature review of spaced-retrieval interventions: a direct memory intervention for people with dementia. *International Psychogeriatrics*.

[28] Graff M. J. L., Vernooij-Dassen M. J. M., Thijssen M., Dekker J., Hoefnagels W. H. L., Rikkert M. G. M. O. (2006). Community based occupational therapy for patients with dementia and their care givers: randomised controlled trial. *BMJ*.

[29] Van’t Leven N., Prick A.-E. J. C., Groenewoud J. G., Roelofs P. D. D. M., de Lange J., Pot A. M. (2013). Dyadic interventions for community-dwelling people with dementia and their family caregivers: a systematic review. *International Psychogeriatrics*.

[30] Piras F., Carbone E., Faggian S., Salvalaio E., Gardini S., Borella E. (2017). Efficacy of cognitive stimulation therapy for older adults with vascular dementia. *Dementia & Neuropsychologia*.

[31] Clare L. (2007). *Neuropsychological Rehabilitation and People with Dementia*.

[32] Clare L., Woods R. T., Linda C., Woods R. T. (2001). *Cognitive Rehabilitation in Dementia: A Special Issue of Neuropsychological Rehabilitation*.

[33] Clare L., Woods R. T., Moniz Cook E. D., Orrell M., Spector A. (2003). Cognitive rehabilitation and cognitive training for early-stage Alzheimer’s disease and vascular dementia. *Cochrane Database of Systematic Reviews*.

[34] Pryor J., Dean S. G., Dean S. G., Siegert R. J., Taylor W. J. (2012). The person in context. *Interprofessional Rehabilitation: A Person-Centred Approach*.

[35] Wade D. (2015). Rehabilitation—a new approach. Overview and part one: the problems. *Clinical Rehabilitation*.

[36] Grant M. J., Booth A. (2009). A typology of reviews: an analysis of 14 review types and associated methodologies. *Health Information & Libraries Journal*.

[37] Liberati A., Douglas G. A., Jennifer T. (2018). The PRISMA statement for reporting systematic reviews and meta-analyses of studies that evaluate health care interventions: explanation and elaboration. *Journal of Clinical Epidemiology*.

[38] Moher D., Liberati A., Tetzlaff J., Altman D. G., The PRISMA Group (2009). Preferred reporting items for systematic reviews and meta-analyses: the PRISMA statement. *Annals of Internal Medicine*.

[39] Egilstrod B., Ravn M. B., Petersen K. S. (2019). Living with a partner with dementia. A systematic review and thematic synthesis of spouses’ lived experiences of changes in their everyday lives. *Aging & Mental Health*.

[40] Guitar N. A., Connelly D. M., Nagamatsu L. S., Orange J. B., Muir-Hunter S. W. (2018). The effects of physical exercise on executive function in community-dwelling older adults living with Alzheimer’s-type dementia: a systematic review. *Ageing Research Reviews*.

[41] World Health Organization *ICF Browser*.

[42] Centre for evidence-based Medicine (2009). Oxford centre for evidence-based medicine—levels of evidence (March 2009). https://www.cebm.net/2009/06/oxford-centre-evidence-based-medicine-levels-evidence-march-2009/.

[43] Nationalt videnscenter for demens (2017). Vurdering af praktisk funktionsevne. http://www.videnscenterfordemens.dk/vaerktoejer/vurdering-af-praktisk-funktionsevne/.

[44] JPND Working Group on Longitudinal Cohorts (2015). *Dementia Outcome Measures: Charting New Territtory*.

[45] Tsuchiya K., Yamaguchi T., Fujita T. (2016). A quasi-randomized controlled trial of brain-activating rehabilitation in an acute hospital. *American Journal of Alzheimer’s Disease & Other Dementiasr*.

[46] Tanaka S., Honda S., Nakano H., Sato Y., Araya K., Yamaguchi H. (2017). Comparison between group and personal rehabilitation for dementia in a geriatric health service facility: single-blinded randomized controlled study. *Psychogeriatrics*.

[47] Werheid K., Köhncke Y., Ziegler M., Kurz A. (2015). Latent change score modeling as a method for analyzing the antidepressant effect of a psychosocial intervention in Alzheimer’s disease. *Psychotherapy and Psychosomatics*.

[48] Brueggen K., Kasper E., Ochmann S. (2017). Cognitive rehabilitation in Alzheimer’s disease: a controlled intervention trial. *Journal of Alzheimer’s Disease*.

[49] Ochmann S., Dyrba M., Grothe M. J. (2017). Does functional connectivity provide a marker for cognitive rehabilitation effects in Alzheimer’s disease? An interventional study. *Journal of Alzheimer’s Disease*.

[50] Salotti P., De Sanctis B., Clementi A., Fernandez Ferreira M., De Silvestris T. (2013). Evaluation of the efficacy of a cognitive rehabilitation treatment on a group of Alzheimer’s patients with moderate cognitive impairment: a pilot study. *Aging Clinical and Experimental Research*.

[51] Thivierge S., Jean L., Simard M. (2014). A randomized cross-over controlled study on cognitive rehabilitation of instrumental activities of daily living in Alzheimer disease. *American Journal of Geriatric Psychiatry*.

[52] Brunelle-Hamann L., Thivierge S., Simard M. (2015). Impact of a cognitive rehabilitation intervention on neuropsychiatric symptoms in mild to moderate Alzheimer’s disease. *Neuropsychological Rehabilitation*.

[53] van Paasschen J., Clare L., Yuen K. S. L. (2013). Cognitive rehabilitation changes memory-related brain activity in people with Alzheimer disease. *Neurorehabilitation and Neural Repair*.

[54] Clare L., Linden D. E. J., Woods R. T. (2010). Goal-oriented cognitive rehabilitation for people with early-stage Alzheimer disease: a single-blind randomized controlled trial of clinical efficacy. *American Journal of Geriatric Psychiatry*.

[55] Kim S. (2015). Cognitive rehabilitation for elderly people with early-stage Alzheimer’s disease. *Journal of Physical Therapy Science*.

[56] Neely A. S., Vikström S., Josephsson S. (2009). Collaborative memory intervention in dementia: caregiver participation matters. *Neuropsychological Rehabilitation*.

[57] Man D., Lee G. Y., Yu E., Yip C. C. (2013). Evaluation of a computer-assisted errorless learning-based memory training program for patients with early Alzheimer’s disease in Hong Kong: a pilot study. *Clinical Interventions in Aging*.

[58] Schwenk M., Zieschang T., Oster P., Hauer K. (2010). Dual-task performances can be improved in patients with dementia: a randomized controlled trial. *Neurology*.

[59] Amieva H., Robert P. H., Grandoulier A.-S. (2016). Group and individual cognitive therapies in Alzheimer’s disease: the ETNA3 randomized trial. *International Psychogeriatrics*.

[60] Regan B., Wells Y., Farrow M., O’Halloran P., Workman B. (2017). MAXCOG–maximizing cognition: a randomized controlled trial of the efficacy of goal-oriented cognitive rehabilitation for people with mild cognitive impairment and early Alzheimer disease. *American Journal of Geriatric Psychiatry*.

[61] Toba K., Nakamura Y., Endo H. (2014). Intensive rehabilitation for dementia improved cognitive function and reduced behavioral disturbance in geriatric health service facilities in Japan. *Geriatrics & Gerontology International*.

[62] Tay L., Lim W. S., Chan M., Ali N., Chong M. S. (2016). A combined cognitive stimulation and physical exercise programme (MINDVital) in early dementia: differential effects on single- and dual-task gait performance. *Gerontology*.

[63] Fernández-Calvo B., Contador I., Ramos F., Olazarán J., Mograbi D. C., Morris R. G. (2015). Effect of unawareness on rehabilitation outcome in a randomised controlled trial of multicomponent intervention for patients with mild Alzheimer’s disease. *Neuropsychological Rehabilitation*.

[64] Viola L. F., Nunes P. V., Yassuda M. S. (2011). Effects of a multidisciplinar cognitive rehabilitation program for patients with mild Alzheimer’s disease. *Clinics*.

[65] Santos G. D., Nunes P. V., Stella F. (2015). Multidisciplinary rehabilitation program: effects of a multimodal intervention for patients with Alzheimer’s disease and cognitive impairment without dementia. *Archives of Clinical Psychiatry (São Paulo)*.

[66] Chew J., Chong M. S., Tay L., Fong Y.-L. (2015). Outcomes of a multimodal cognitive and physical rehabilitation program for persons with mild dementia and their caregivers: a goal-oriented approach. *Clinical interventions in aging*.

[67] Onor M. L., Trevisiol M., Negro C., Alessandra S., Saina M., Aguglia E. (2007). Impact of a multimodal rehabilitative intervention on demented patients and their caregivers. *American Journal of Alzheimer’s Disease & Other Dementiasr*.

[68] Särkämö T., Tervaniemi M., Laitinen S. (2014). Cognitive, emotional, and social benefits of regular musical activities in early dementia: randomized controlled study. *The Gerontologist*.

[69] Laakkonen M.-L., Kautiainen H., Hölttä E. (2016). Effects of self-management groups for people with dementia and their spouses-randomized controlled trial. *Journal of the American Geriatrics Society*.

[70] Schiffczyk C., Romero B., Jonas C., Lahmeyer C., Müller F., Riepe M. W. (2013). Efficacy of short-term inpatient rehabilitation for dementia patients and caregivers: prospective cohort study. *Dementia and Geriatric Cognitive Disorders*.

[71] Dean S. G., Siegert R. J., Taylor W. J., Dean S. G., Siegert R. J., Taylor W. J. (2012). Conclusion: rethinking rehabilitation. *Interprofessional Rehabilitation: a Person-Centred Approach*.

[72] Leplege A., Gzil F., Cammelli M., Lefeve C., Pachoud B., Ville I. (2007). Person-centredness: conceptual and historical perspectives. *Disability and rehabilitation*.

[73] Kitwood T. (1997). *Dementia Reconsidered: The Person Comes First*.

[74] Wade D. (2016). Rehabilitation—a new approach. part four: a new paradigm, and its implications. *Clinical Rehabilitation*.

[75] Sundhedsstyrelsen (2016). *Livet Med Demens—Styrket Kvalitet i Indsatsen: Fagligt Oplæg Til den Nationale Demenshandlingsplan 2025*.

[76] Gitlin L. N., Corcoran M., Winter L., Boyce A., Hauck W. W. (2001). A randomized, controlled trial of a home environmental intervention. *The Gerontologist*.

[77] Bachmann S., Finger C., Huss A., Egger M., Stuck A. E., Clough-Gorr K. M. (2010). Inpatient rehabilitation specifically designed for geriatric patients: systematic review and meta-analysis of randomised controlled trials. *BMJ*.

[78] Momsen A., Rasmussen J., Nielsen C., Iversen M., Lund H. (2012). Multidisciplinary team care in rehabilitation: an overview of reviews. *Journal of Rehabilitation Medicine*.

[79] Buss D. DAISY—Hverdagen med Demens.

[80] McKhann G. M., Knopman D. S., Chertkow H. (2011). The diagnosis of dementia due to Alzheimer’s disease: recommendations from the National Institute on Aging-Alzheimer’s association workgroups on diagnostic guidelines for Alzheimer’s disease. *Alzheimer’s and Dementia*.

[81] Koretz R. L. (2019). Assessing the evidence in evidence-based medicine. *Nutrition in Clinical Practice*.

[82] Ávila R., Carvalho I. A. M., Bottino C. M. C., Miotto E. C. (2007). Neuropsychological rehabilitation in mild and moderate Alzheimer’s disease patients. *Behavioural Neurology*.

[83] Kurz A., Thöne-Otto A., Cramer B. (2012). Cordial. *Alzheimer Disease & Associated Disorders*.

[84] Hattink B. J., Meiland F. J., Overmars-Marx T. (2016). The electronic, personalizable Rosetta system for dementia care: exploring the user-friendliness, usefulness and impact. *Disability and Rehabilitation Assistive Technology*.

[85] Bettcher B. M., Giovannetti T., Libon D. J., Eppig J., Wambach D., Klobusicky E. (2011). Improving everyday error detection, one picture at a time: a performance-based study of everyday task training. *Neuropsychology*.

[86] Nationalt videnscenter for demens (2016). Aktivitet i hverdagen. http://www.videnscenterfordemens.dk/pleje-og-behandling/pleje-og-omsorg/aktivitet-i-hverdagen/.

[87] Sobol N. A., Hoffmann K., Vogel A. (2016). Associations between physical function, dual-task performance and cognition in patients with mild Alzheimer’s disease. *Aging & Mental Health*.

